# An Assistive Technology System that Provides Personalized Dressing Support for People Living with Dementia: Capability Study

**DOI:** 10.2196/medinform.5587

**Published:** 2018-05-01

**Authors:** Winslow Burleson, Cecil Lozano, Vijay Ravishankar, Jisoo Lee, Diane Mahoney

**Affiliations:** ^1^ NYU-X College of Nursing New York University New York, NY United States; ^2^ Motivational Environments Research Group School of Computing, Informatics, and Decision Systems Engineering Arizona State University Tempe, AZ United States; ^3^ School of Nursing MGH Institute of Health Professions Charlestown, MA United States

**Keywords:** Alzheimer disease, disorders, neurocognitive, image processing, computer-assisted

## Abstract

**Background:**

Individuals living with advancing stages of dementia (persons with dementia, PWDs) or other cognitive disorders do not have the luxury of remembering how to perform basic day-to-day activities, which in turn makes them increasingly dependent on the assistance of caregivers. Dressing is one of the most common and stressful activities provided by caregivers because of its complexity and privacy challenges posed during the process.

**Objective:**

In preparation for in-home trials with PWDs, the aim of this study was to develop and evaluate a prototype intelligent system, the DRESS prototype, to assess its ability to provide automated assistance with dressing that can afford independence and privacy to individual PWDs and potentially provide additional freedom to their caregivers (family members and professionals).

**Methods:**

This laboratory study evaluated the DRESS prototype’s capacity to detect dressing events. These events were engaged in by 11 healthy participants simulating common correct and incorrect dressing scenarios. The events ranged from donning a shirt and pants inside out or backwards to partial dressing—typical issues that challenge a PWD and their caregivers.

**Results:**

A set of expected detections for correct dressing was prepared via video analysis of all participants’ dressing behaviors. In the initial phases of donning either shirts or pants, the DRESS prototype missed only 4 out of 388 expected detections. The prototype’s ability to recognize other missing detections varied across conditions. There were also some unexpected detections such as detection of the inside of a shirt as it was being put on. Throughout the study, detection of dressing events was adversely affected by the relatively smaller effective size of the markers at greater distances. Although the DRESS prototype incorrectly identified 10 of 22 cases for shirts, the prototype preformed significantly better for pants, incorrectly identifying only 5 of 22 cases. Further analyses identified opportunities to improve the DRESS prototype’s reliability, including increasing the size of markers, minimizing garment folding or occlusions, and optimal positioning of participants with respect to the DRESS prototype.

**Conclusions:**

This study demonstrates the ability to detect clothing orientation and position and infer current state of dressing using a combination of sensors, intelligent software, and barcode tracking. With improvements identified by this study, the DRESS prototype has the potential to provide a viable option to provide automated dressing support to assist PWDs in maintaining their independence and privacy, while potentially providing their caregivers with the much-needed respite.

## Introduction

### Background

Dementia is a term that describes a broad category of symptoms related to declining memory and eroding thinking skills. It is a syndrome associated with a number of progressive illnesses affecting memory, thinking, behavior, and the ability to perform everyday activities [1]. An estimated two-thirds of dementia cases may be due to Alzheimer disease, the most common form of dementia [2]. The second most common cause is vascular dementia due to stroke. The World Health Organization Report estimates 7.7 million new cases of dementia are diagnosed every year [3] and the current estimate of cases to be 47.5 million. Over the next 40 years, an estimated 682 million people will live with dementia (persons with dementia, PWDs) [1]. It is important to note that the cognitive declines experienced because of dementia are not a normal part of the aging process. Beyond memory, dementia can affect communication and understanding, as well as the ability to focus and make decisions.

Dementia and cognitive decline make both basic and instrumental activities of daily living (ADL), such as bathing, dressing, eating, paying bills, cleaning, and doing laundry difficult and challenging for the individuals and their caregivers. Almost every person diagnosed with dementia eventually must either rely extensively on a caregiver (often a family member) at his or her home or relocate into a nursing home for professional supplemental care.

Data indicate that 86% of caregivers (either family members or professionals) help with ADL activities; the most common being dressing 61%, followed by feeding 52%, bathing 37%, toileting 34%, and incontinence care 26% [4]. Caregivers assisting individuals with dementia who perform ADL often feel stressed, frustrated at the amount of time required, and emotionally challenged. For individuals with early to middle stage dementia, dressing has been reported as the most pressing concern for both patients and caregivers [5]. Core challenges of dressing include the complexity of the activity and issues of privacy and independence of the PWD, particularly when caregivers are family members. Data indicate that adult children find it particularly challenging to help dress their parents, especially for opposite genders [6].

Our semiautonomous DRESS prototype was designed to address some of the challenges associated with dressing identified in literature and from interaction with focus groups composed of family and professional PWD caregivers. Integrating automated tracking and recognition with guided assistance for the dressing process, the DRESS prototype uses a combination of sensors and image recognition to detect dressing states and embedded intelligence to guide and prompt dressing tasks, assist in correction of dressing errors, and provide reinforcement for positive dressing performance. Initial input from caregiver focus groups provided a foundation for design and development of the prototype [6].

The goal was to provide assistance for the individual PWD to help them age in place more gracefully, while ideally allowing the caregiver to do other tasks as the PWD dresses, with the assurance that the prototype will monitor and alert when the dressing process is completed, or prompt if an intervention is needed. Although attempts have been made to automate real-time assistance for routine activities, we are not aware of other context-aware computing and human-computer-interaction projects that incorporate real-time prompting to assist with dressing processes.

The DRESS prototype leverages computer vision-based technologies to track and recognize progress during the dressing process. reacTIVision [7] is an image recognition system that the DRESS prototype uses to track fiducial markers [8] (a type of barcode, see Dressing Event Detection section) imprinted on clothing items (shirts and pants) to identify the type, location, and orientation of a garment. DRESS uses this data to recognize and track user progress while dressing and to determine whether the clothing is correctly positioned and oriented (ie, the front of the pants is facing forward). When a dressing error is detected, such as putting pants on backwards, the prototype generates an appropriate audio prompt recorded in the caregiver’s voice to inform the PWD, noting the nature of the mistake and prompting recovery actions to correct the mistake. Once each step has been completed correctly, DRESS provides feedback and prompts the PWD to progress to the next step of the dressing activity. If the PWD continues to have problems—“freezing” or making little or no progress after multiple attempts or becoming frustrated—the caregiver is alerted so that they may provide personal assistance and support.

Before deploying the DRESS prototype in real-world environments with PWDs, we ran a capabilities study. Capabilities studies are employed in engineering to ensure that processes or products meet customer requirements, specifications, and functionality metrics. Our capabilities study involved 11 healthy participants emulating common dementia dressing scenarios, with a shirt and pants [9], to evaluate the DRESS prototype.

### Prior Work

Many assistive technologies have been developed to help people perform daily activities. However, few systems specifically target the needs of PWDs. Mihailidis et al [10] developed a system for persons with moderate to severe dementia to assist with handwashing. Examples of other targeted behaviors include cooking [11] and taking medications [12]. Literature supports the use of cognitive interventions to assist and improve individual ability to perform ADL [9]. The current state-of-the-art in technology interventions involves attempts to mathematically predict patterns of behavior, but the results to date have been criticized as disappointing, as the predictive results are poor, and they are not sufficiently capable to be relevant to the design of systems that support the needs of PWDs [13-18].

Context-aware memory aids may have the potential to provide the support needed to assist with daily activities such as prompting to start dressing processes. However, memory aids alone are not sufficient—it is also important to determine context (current stage of dressing) to be able to create effective prompts. Efficient activity recognition systems are needed to acquire this information.

### Usefulness of Vision Systems

Wu et al [19] presented an activity recognition system that combined radio-frequency identification (RFID) and video feedback in a kitchen setting. In testing for 16 activities with 33 subjects, they achieved a recognition rate of 80%. The system developed by Mihailidis et al [10] used video processing to recognize context and prompt actions performed in handwashing.

### Behavioral Identification

An important contribution in the advancement of systems that identify ADL is Proact [20], a project designed to address recognition of 14 everyday activities. The system reports both the activity being performed and the extent to which it is performed. Related ADL research has been advanced by Dalton et al [21] and Fleury et al [22]. Dalton et al [21] developed a system that uses wireless kinematic sensors to identify accuracy of ADL identification based on position and on the manner of data processing. The authors reported dressing among the activities recognized. Fleury et al [22] developed a system using support vector machines and the data acquired from infrared sensors, microphones, door contact sensors, webcams, and accelerometers to recognize when a subject performs six types of daily activities, including dressing and undressing. Hayes et al [12] present another example of context-aware prompted feedback in an electronic pillbox that continuously monitors medication-taking over time.

### Dementia Dressing Work

We found that little research emphasis has been placed on developing dressing support technologies to assist PWDs with this important ADL. An early effort in assisting PWDs in dressing was conducted by Namazi and Johnson [23]. They demonstrated how modifying closet arrangement, to organize the clothing in a visible and preplanned sequential order, can help independent activity.

Engelman et al [24] showed how human prompting using graduated procedures can be used to increase dressing independence for PWDs. Popleteev and Mayora [25] developed a smart assistive buttoning system for people with mild cognitive decline. Their system detects whether a button is “locked” with its correct counterpart and if incorrect (unlocked or locked with a wrong counterpart), triggers an event, and the system provides an alert (verbal feedback) to the user and records event details for further caregiver analysis.

Matic et al [26] developed an RFID and video system that detects dressing activity failures. Their system identifies the most common dressing failures, which are as follows: (1) putting clothes in an incorrect order, (2) putting on clothes partially, (3) incorrect orientation (such as putting on clothes backwards), (4) dressing incorrectly for temperature (too little or to many layers of clothing), and (5) putting clothes on the wrong part of the body. In addition to identifying errors, Matic’s system also recognizes when the correct dressing performance has occurred. However, this system does not use this information to provide feedback or to assist in rectifying mistakes identified during dressing.

Although each of these systems provides significant contributions, none are comprehensive in a manner that addresses the entire process from monitoring dressing activity, to identifying correct dressing and dressing failures, to providing feedback and guidance that rectify mistakes.

An important and challenging feature missing in existing systems is the ability to tailor or customize feedback and support interventions that address varying levels of cognitive function in an individual PWD. Cognitive function can progressively decline over time at various rates fluctuating throughout the day or over time as the system is used (eg, weeks to months or years) [9,13].

Literature and caregiver focus groups [27], consisting of 25 Latino (family member and professional) caregivers of PWDs, in three Arizona community service sites serving Latinos, clearly indicate an effective dressing assistance system for people with dementia should aim to be: (1) unobtrusive, (2) automated, (3) context-aware (ie, having the ability to recognize actions performed or missed), and (4) capable of providing personally tailored feedback and assistance as needed. The DRESS prototype was developed with attention to each of these criteria. The results, discussed below, indicate the potential of automated systems to assist in actual independent and assisted living settings.

### The DRESS Prototype

**Figure 1:**
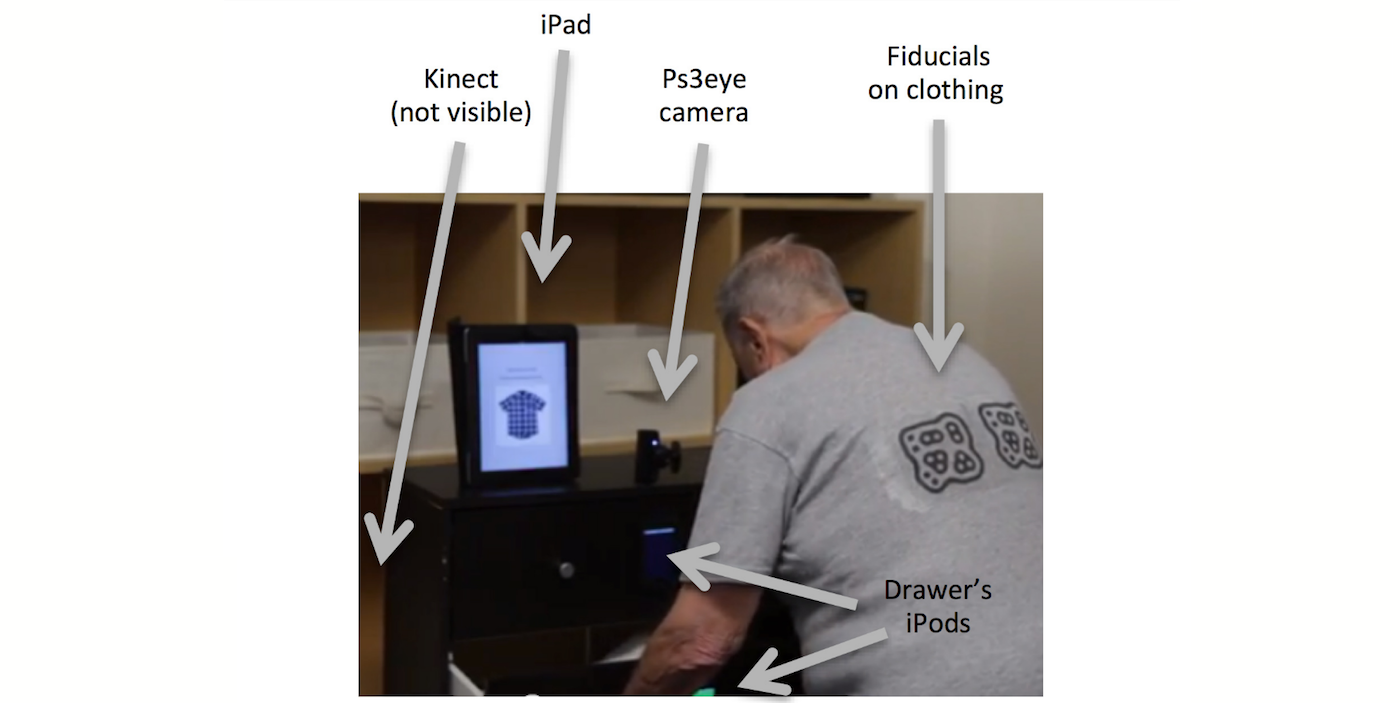
Typical human interaction with the DRESS prototype.(9, reprinted with permission).

**Figure 2:**
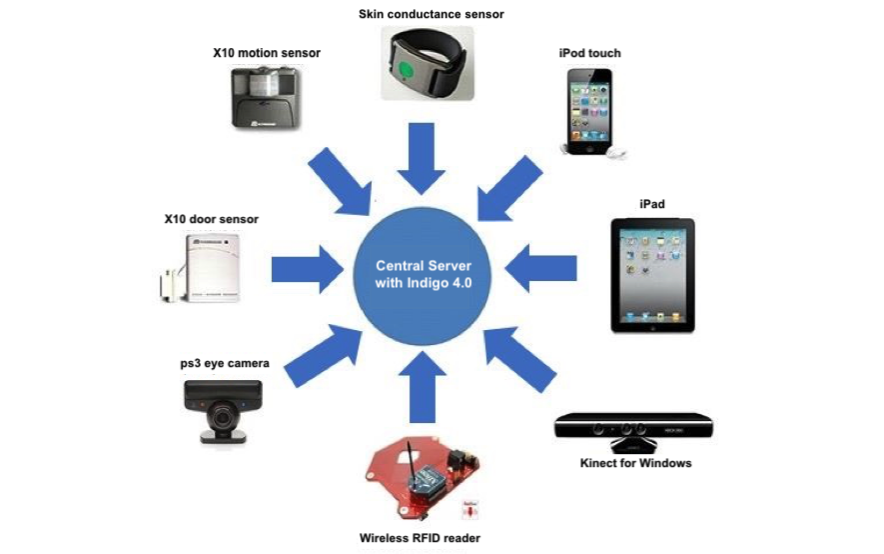
DRESS prototype initial architecture hardware and infrastructure; Kinect was subsequently removed.

**Figure 3:**
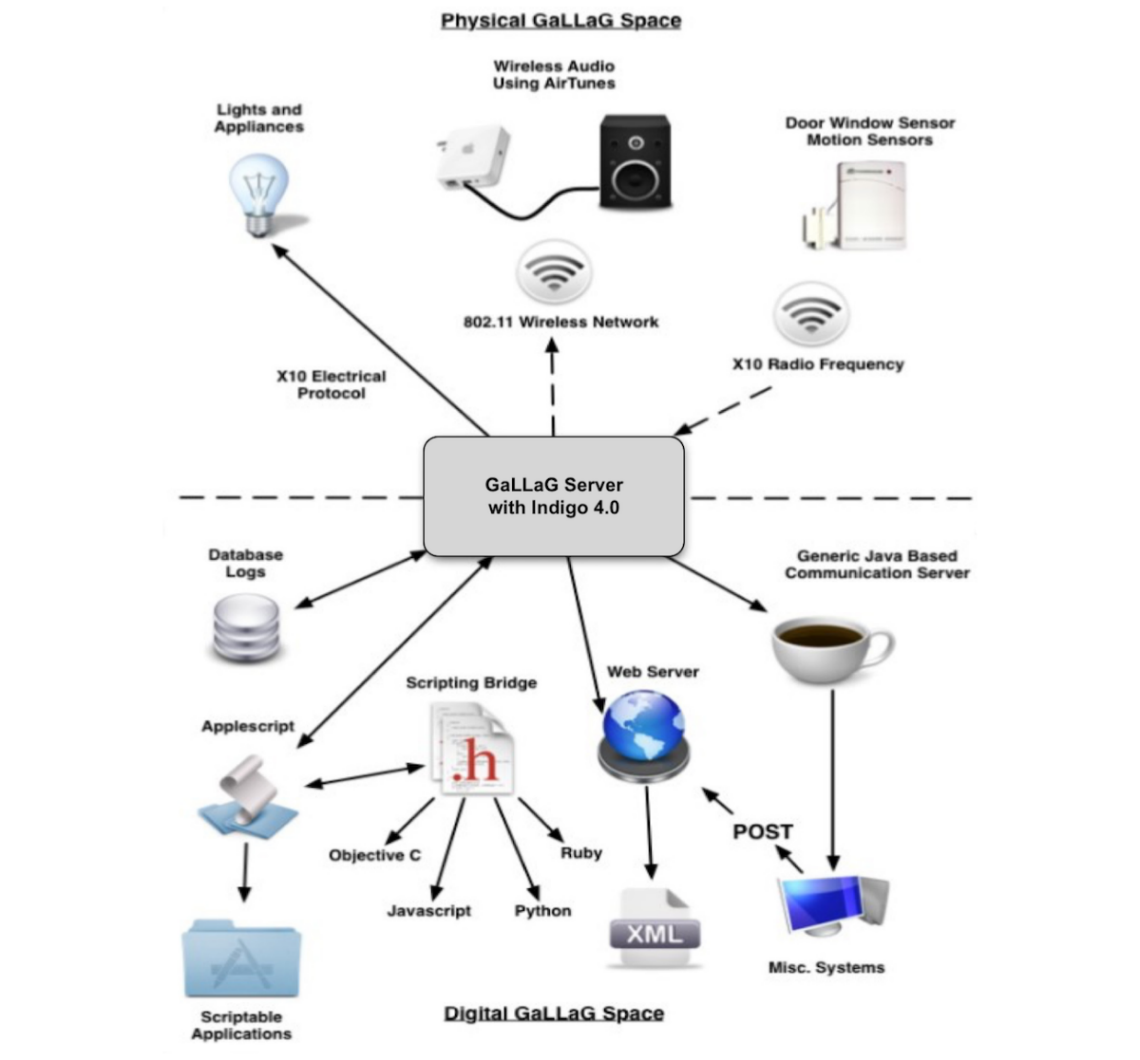
Game as Life—Life as Game system architecture including physical components (lighting using X10 electrical protocol, wireless audio using AirTunes and an 802.11 wireless network, and door window sensor using X10 radio frequency) above the horizon and digital components (Java server, web, database logs, and scripts).

The transdisciplinary team that developed the DRESS prototype included experts from engineering, gerontology, social science, psychology, nursing, speech, and occupational rehabilitation. The DRESS prototype was advanced through an iterative user-centered design approach, integrating participation and involvement of caregiver teams and family members of PWDs in the process of problem definition, design, and development [9] (Figures 1 and 2). Design was also informed by the Alzheimer’s Association’s dressing guidelines. The DRESS prototype uses our Game as Life-Life as Game (Figure 3) ubiquitous computing platform [9] that integrates a variety of sensors with digital systems, databases, and interactive software scripting to analyze context, then to create and provide prompts and interactions to support PWDs and caregiver’s dressing-related activities.

The intent of the DRESS prototype is to integrate typical routines and humanized interactions, promote normalcy and safety, and facilitate flexible customization to guide PWDs through dressing processes [9]. Continuous collection of data offers the DRESS prototype unique opportunities to not only provide real-time guidance and feedback but also to record, analyze, and understand patterns of usage to enhance development of appropriate interventions. We focused on tailoring and customizing feedback to suit each user’s need and also recognized the progressive nature of dementia so that the prototype focuses on the individual, not on the disease. The DRESS prototype is further customizable to use a recording of the caregiver’s voice to prompt the PWD.

The DRESS prototype also analyzes the marker data to determine which portion of the clothing is facing the dresser. Caregiver focus groups [9] indicated that this level of privacy was acceptable to (both professional and family) caregivers, but they recommended that future versions of the DRESS prototype use cameras embedded into the surface of the dresser, such that their visibility to PWDs would be minimized. Likewise, in future DRESS prototypes, we plan to make the coded markers (which are currently large, awkward, and would likely be stigmatizing, if used in public) “invisible,” either through the use of infrared ink or through machine recognition and training of patterns in PWDs’ existing clothes (see Discussion, for additional information).

### DRESS Functionality Aiding Dressing

The DRESS scenario begins with a caregiver assisting the PWD, who is wearing underwear and the wrist or leg skin conductance sensor, positioned in front of the dresser. Following the Alzheimer’s Association’s dressing guidelines for logical ordering and simplification of clothing choices, the five-drawer dresser is organized with one piece of clothing per drawer, with the first clothing item in the top drawer and the remaining clothing items sequenced in drawers below. The caregiver then initiates the assistive dressing procedure via their mobile device and leaves the room.

An X10 motion sensor on top of the dresser senses that the PWD is close to the dresser and transmits status to the Indigo server. Once the DRESS prototype confirms the presence of the PWD, the individual receives a verbal prompt to open the top drawer, and the iPod Touch on the front of the drawer displays a green light prompt. The other drawers display a red light. If the PWD opens the wrong drawer, the DRESS prototype prompts the PWD to close it and open the drawer with the green light (ie, the correct drawer).

Once the PWD opens the correct drawer and removes the clothing item, the RFID reader inside the drawer detects movement of the tag attached to the clothing. When this occurs, the DRESS prototype initiates a sequence, beginning with an Indigo action command (developed in the Processing language [7]) for the open source reacTIVision computer vision software to receive fiducial marker data from the cameras. reacTIVision provides information about the orientation and distance of the clothing-based fiducial markers. The Processing program initially used the fiducial marker data in combination with skeletal position data from Kinect for Windows [28] (responsible for skeletal tracking) to identify the current state of dressing, assess the need for intervention, and to provide audio prompts and assistance if needed. As the reacTIVision system captured the most important elements of clothing orientation, it was determined that Kinect was not needed and that the DRESS prototype would be simpler without this component.

**Figure 4:**
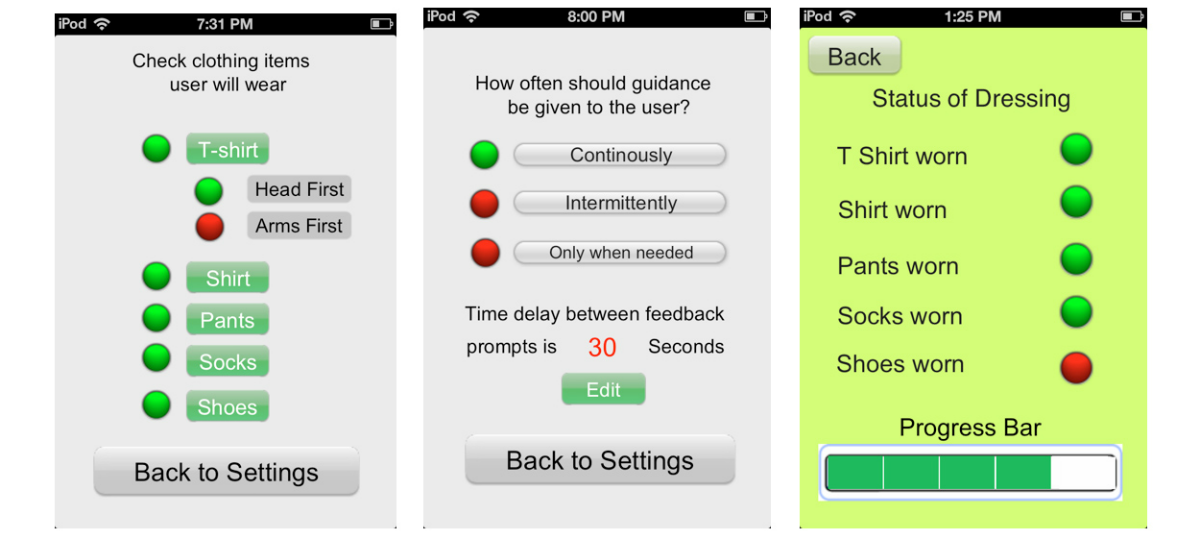
Caregiver iOS mobile application user interface.

If caregiver selects “continuous” mode (see Figure 4, middle) or when the DRESS prototype determines the need for continuous intervention, chronological directions for each step of the dressing process are provided (eg, “put one arm through one hole of the t-shirt” followed by “Now, put the other arm through the other hole.”). If the clothing is sensed as incorrectly worn (eg, inside out, back to front, and shirt Velcro misaligned) or the PWD is not taking any actions, the DRESS prototype identifies this state and guides the PWD through the process of correcting the error through a series of audio and visual prompts.

The DRESS prototype then continues to monitor, sense, and correct the PWD until the dressing process is completed. To enhance autonomy and independence of the PWD, the DRESS prototype does not provide audio prompts when an article of clothing is donned correctly in the “independent” mode. The goal is for the DRESS prototype to personally tailor support, at a level commensurate with the PWD’s varying moment-to-moment and day-to-day needs.

Once the first clothing item is identified as being worn correctly, the DRESS prototype asks the PWD to close the drawer. Upon confirmation of drawer closure, the Indigo server initiates the next step in the dressing sequence, repeating a similar sequence of actions for each item of clothing in the remaining drawers until dressing is completed.

If the PWD becomes “stuck” (a nonoptimal experience state, eg, the user becomes confused or loses interest or focus) [29], as determined by a combination of the skin conductance level and context of recent sequence of problems in dressing activity, motivational prompting is provided to reengage the PWD.

The DRESS prototype continually monitors PWD stress levels via the skin conductance sensor, which is coupled with action monitoring to track progress and tailor interventions to mitigate frustration. If PWD stress levels continue to increase, the DRESS prototype initiates an activity previously identified by the caregiver as soothing, such as playing a favorite song or video clip. If this intervention is unsuccessful and stress levels continue to rise, the DRESS prototype notifies the caregiver via preferred communication (ie, cell phone or email). The goal is to avoid what caregivers term a “meltdown” situation.

The DRESS prototype continues to monitor progress, provide guided prompts and intervention until the dressing process is completed and then notifies the caregiver.

### Dressing Event Detection

**Figure 5:**
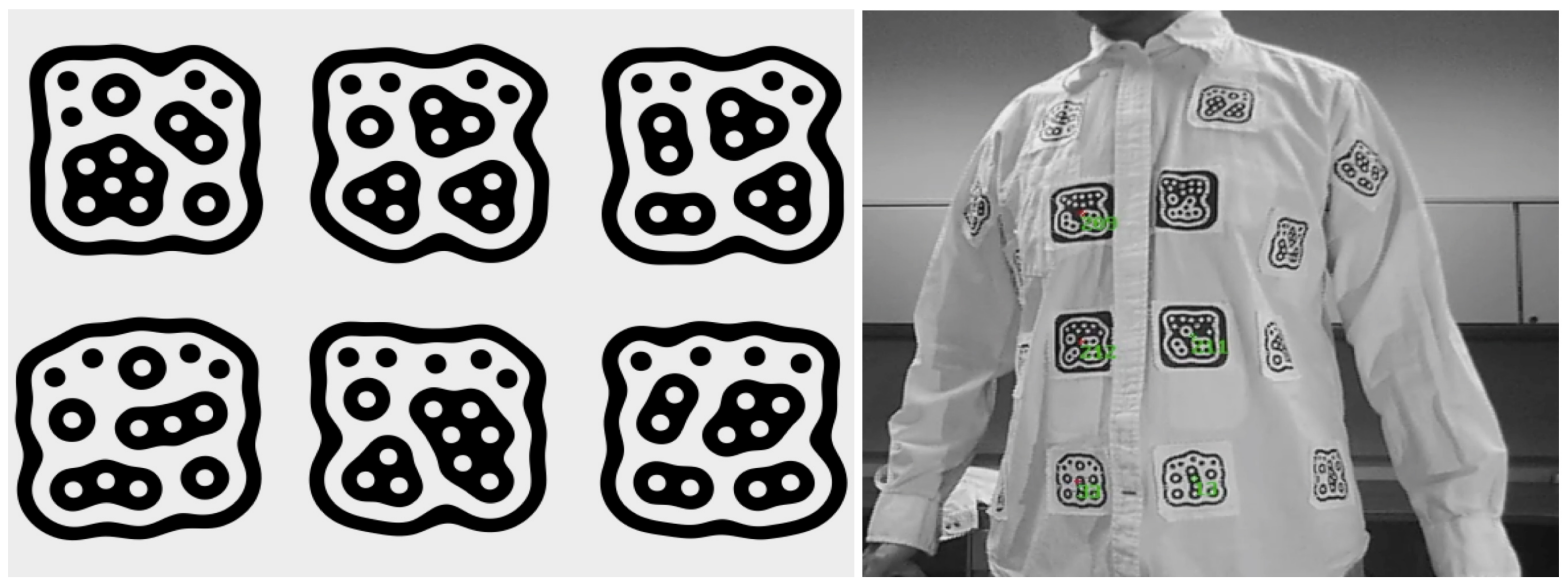
Fiducial markers (left) and example of fiducial markers (right) provided by a view of the ps3eye camera showing the markers from the shirt detected by the reacTIVision system.

The fiducial tracking system uses a ps3eye camera to capture the current state of the shirt dressing process by detecting the ID and position of the specially designed 2D bar code fiducial markers attached to the clothing (Figure 5). The reacTIVision software extracts marker identification information from the cameras (identifiers indicate that the system detected the markers; see Figure 5, right), sensing tangible user interface object messages via UDP 3333 to the tangible user interface object–enabled client application [8]. This software determines (1) the dressing condition scenario based on the marker position with respect to the clothing, (2) its orientation and relation with other markers, (3) the time the marker is detected, and (4) the context of the dressing process. Table 1 shows the event detection and the rules used for the process of donning a shirt and a pair of pants.

The reacTIVision recognition process begins by searching for any marker within the camera field of view. reacTIVision uses marker data to determine orientation and placement of the garment. For example, sensing the back or incorrect positioning of the shirt prompts the PWD to reorient the shirt. Sensing the left or right side of the shirt marker can determine whether the user has put one side of the shirt on correctly (F and R/L in Table 1). Subsequent sensing of the markers on the opposite side (L/R) can indicate that both arms are worn (A). If the markers indicating reorientation of progress have not been sensed for more than 5 seconds, the DRESS prototype interprets this condition as partial dressing (p) and provides additional guidance and prompts.

**Table 1:** Detection event descriptions and identification rules.

Clothing item^a^		Detection description	Identification rules
**Shirt**			
	F	Front side of the shirt	Any marker from front of shirt (5,6,10-14,30-34,208,209,211,212) is visible for 2+ seconds.
	B	Back side of the shirt	Any marker from back of shirt (7) is visible.
	I	Inside part of the shirt	Any marker from inside of shirt (8,9) is visible for 2+ seconds. Marker 8 is for the center part of inside, whereas marker 9 is for the sides (left and right) of the inside part.
	R	Right arm of the shirt worn	Any markers from front right of shirt (5,10,11,12,13,14,208,211) are visible for 2+ seconds.
	L	Left arm of the shirt worn	Any markers from front left of shirt (6,30,31,32,33,34,209,212) are visible for 2+ seconds.
	A	Both arms of the shirt worn	Any one marker from detecting R and one for detecting L is visible for 2+ seconds.
	M	Velcro unevenly fastened	Any one of the following absolute differences of markers’ distances are true: |Y208-Y209| >.05, |Y211-Y212| >.05, |X208-X209| >.18, or |X211-X212| >.18.
	p	Partial dressing (incomplete)	Any one of the markers from either L or R is visible and the other is not visible for more than 5 seconds.
	C	Shirt worn correctly	All the following absolute differences of markers’ distances are true: |Y208-Y209| <.05, |Y211-Y212| <.05, |X208-X209| <.18, and |X211-X212| <.18.
**Pants**			
	B	Back side of the pants	Any marker from back of pants (22) is visible for 2+ seconds.
	I	Inside part of the pants	Any marker from inside of pants (17,19) is visible for 2+ seconds. Marker 17 is for inside out and front side, and marker 19 is for inside out and back side.
	L	Left leg of the pants worn	Any markers from low part of the left side of the pants (28,29) are visible for 2+ seconds.
	R	Right leg of the pants worn	Any markers from low part of the right side of the pants (25,26) are visible for 2+ seconds.
	p	Partial dressing (incomplete)	Any one of the markers from either L or R is visible and the other is not visible for more than 5 seconds.
	C	Pants worn correctly	All markers from upper part of the front of pants (15,16,24,27) are visible for 2+ seconds.

^a^The letters in this column indicate the ID.

**Figure 6:**
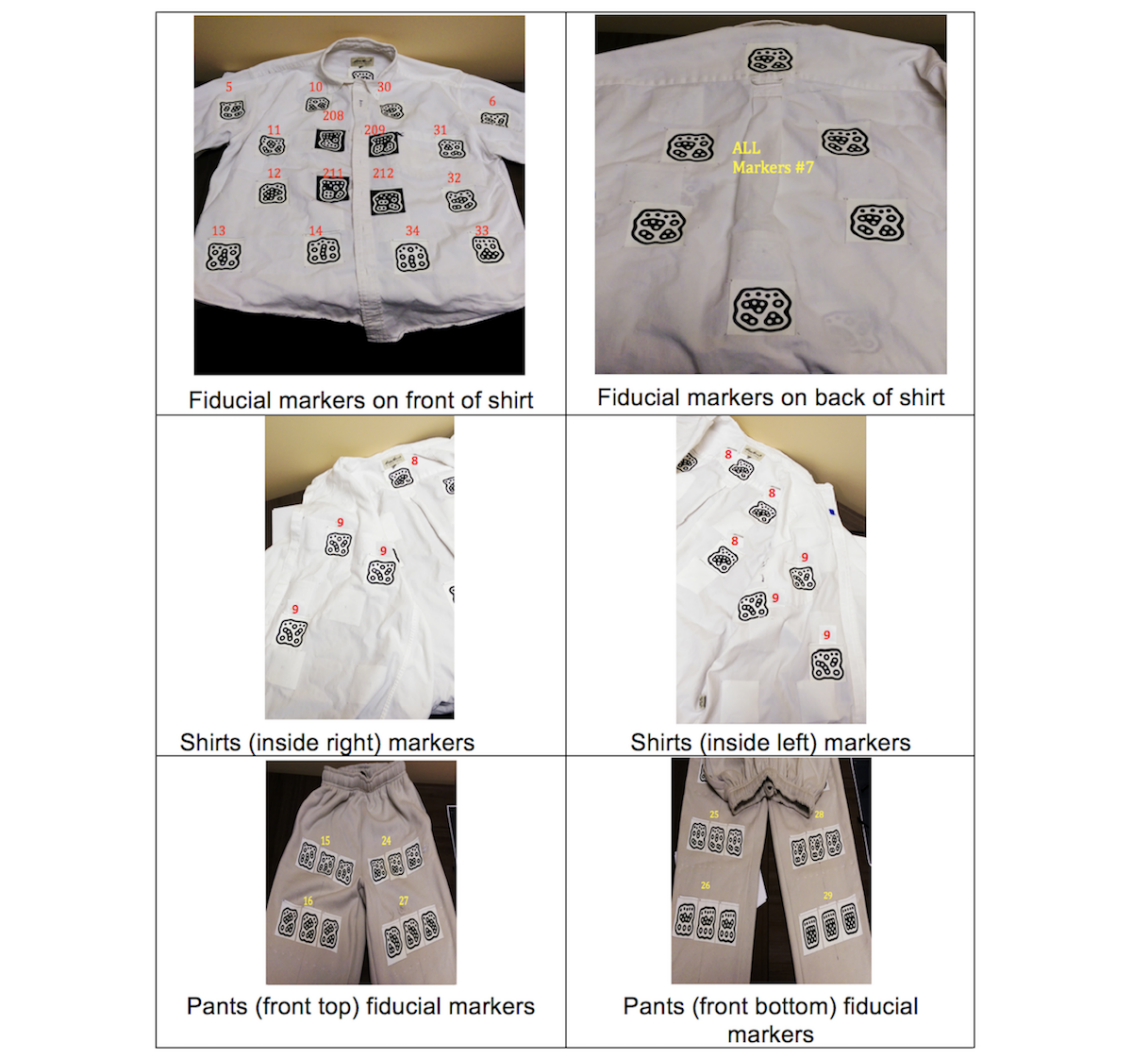
Fiducial markers provide DRESS with unique identification, orientation, and distance information on (a) shirt (upper row); (b) inside right and inside left markers (middle row); and (c) pants (lower row).

To verify that the shirt is closed and worn correctly (C), the DRESS prototype searches for four markers (208/209 and 211/212) placed near the Velcro (Figure 6, top left). The close proximity between the right matching markers of both sides of the shirt and their orientation is used to identify any misalignment and generate corrective prompts. The proximity threshold between matching markers is fixed and previously determined by testing a correctly worn shirt. If the alignment conditions are not met, the DRESS prototype indicates a misalignment error (M) and prompts corrective action.

Dressing errors such as wearing the shirt back to front or inside out are identified if markers are sensed in the wrong sequence or orientation. For example, markers 7, 8, and 9 are attached to the back (B) and inside parts of the shirts (I) to identify the correspondent errors cases (see Figure 6, top row, right, and middle row). During laboratory testing, more than one marker of the same orientation ID was placed on each part of the clothing to assess validity and increase the robustness of the DRESS prototype’s marker detection process.

Testing indicated that while donning the shirt, markers on the back or the inside can inadvertently become sensed. To minimize inaccurate detection errors, the DRESS prototype requires each marker to be continuously visible for a period of time before initiating action. On the basis of observations from our preliminary study [9], a 3-second duration appeared to be adequate to minimize these inaccurate detections.

Once the shirt donning has been successfully completed, the DRESS prototype prompts the PWD to close the shirt drawer, then provides audio and visual cues (green light on the associated iPod) to proceed to the pants drawer. For the pants dressing process, the DRESS prototype uses only the ps3eye camera located in the middle of the dresser. The DRESS prototype begins searching for the fiducial markers on the bottom half of the pants (25/26 and 28/29; see bottom right of Figure 6) to determine orientation and position. Similar to the process used to determine shirt orientation, the DRESS prototype then searches for markers indicating that one leg is worn (R/L) and then the other leg marker (L/R) is worn.

Partial or error detection follows the same protocol as the shirt. To identify whether the user has indeed stood, pulled the pants up, and worn them correctly (C), the DRESS prototype looks for markers in the upper half of the pants, specifically for marker pairs 15/16 and 24/27 (Figure 6, bottom left). This pant detection pattern was based on preliminary study observations of donning the pants while seated (PWDs are generally encouraged to don pants while seated, for safety, to minimize falls). As in the example of the shirt, errors like reversal back to front (B) or inside out (I) are detected with markers 22 and 17/19 attached to these parts of the clothing, and appropriate corrective guidance and prompts are generated for the PWD.

Once the bottom markers of both legs have been detected, the DRESS prototype assumes that the user has donned both the legs of the pants correctly and is about to stand up. Supportive feedback is given and the caregiver is notified that the dressing process has been completed.

## Methods

### Aims

A laboratory study was conducted to evaluate the detection capabilities of the DRESS prototype. The study and recruitment of subjects was conducted according to the ASU IRB protocol STUDY1110006934. No conflicts of interest were noted. As the study was not a clinical trial, it was not registered with Clinicaltrials.gov.

Our overall aim for the capabilities study was to assess the DRESS prototype’s ability to detect correct and incorrect dressing events. Accurate detection of these events is critical to developing effective sociotechnical systems that support a PWD’s dressing activities. Although verification of our findings will be necessary with PWDs in home-based settings, home trials were not feasible during this phase of research. As the primary difference between a healthy adult and a PWD is a cognitive difference, the recruitment of healthy adults, of any age, was considered reasonable for this study.

To ensure the appropriateness of the prototype for PWDs, we built upon our prior work, supported by the Alzheimer’s Association, involving caregiver focus groups [6], with the aim of advancing the prototype’s ability to tailor customized support and feedback to the challenges that PWDs encounter as their conditions progress. We followed caregiver recommendations to engage adult participants in a laboratory setting to produce “acted errors” during DRESS prototype development and testing to assess the DRESS prototype’s ability to accurately detect orientation, position, and errors.

To mitigate privacy and sensitivity issues related to dressing, study participant dressing actions were performed by donning fiducially-enhanced clothes on top of existing tee-shirt and athletic shorts. This study focused only on fiducial detection, and other sensor data were not included. We chose to test two clothing items: shirt and pants because of the requirement for different levels of motion and dexterity for both the top and bottom of the body. In addition, both clothing items are commonly worn by both women and men.

### Study Design

The study was conducted with 11 healthy young participants, engaged for 1-hour sessions (7 female and 4 male, aged 19-41years—average age 25 years). Given the innovative nature of the prototype, we found that there was sufficient participant interest in the study to proceed effectively without the need for compensation. At the beginning of the session, participants gave informed consent, filled out a survey instrument, and were informed of the research goal. Pre- and postsurveys contained questions related to common dressing practices (eg, How often they use the specific clothing item? How often they put the clothing the wrong way? and if any, what have been the most common dressing mistakes they had made?) and reporting any discomfort about both the experimental setting and the tasks.

**Figure 7:**
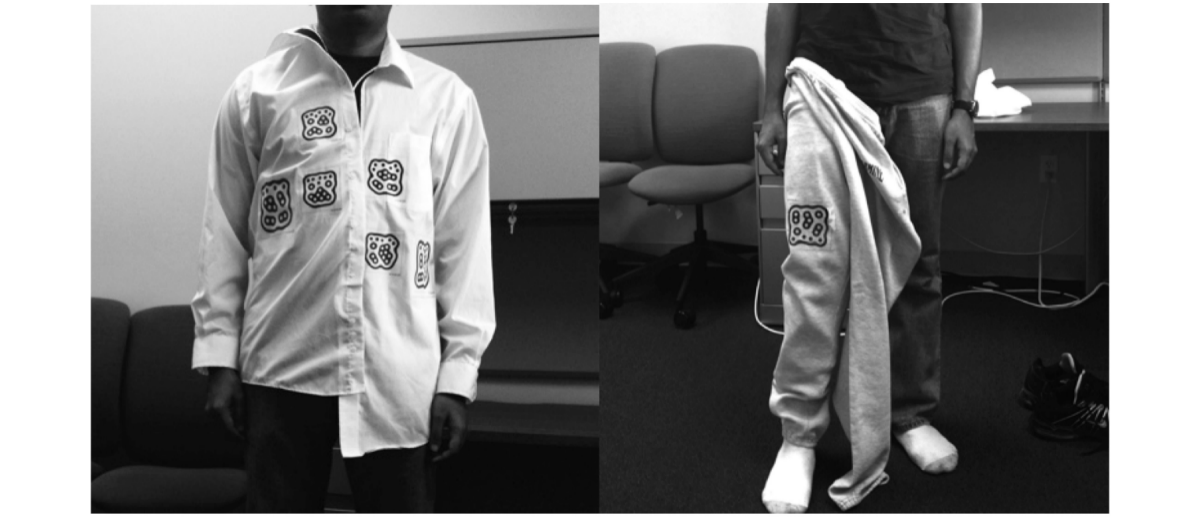
Example pictures of dressing conditions shown to the participants, “Shirt misaligned” (left), “Pants partial” (right).

After the presurvey, participants were introduced to the DRESS prototype, highlighting the location of the fiducial tracking cameras and sensors and the preferred space between the dresser and the chair to use while getting dressed. Subjects were also asked to emulate common target population dressing practice and to use the chair while donning pants for the different dressing scenario study conditions. Finally, subjects were shown the special characteristics of the clothing items to be worn, especially the fact that they had to correctly align the shirt’s Velcro closure without buttons or button holes to assist with alignment (Figure 7).

We were specifically interested in observing the ability of the prototype to use fiducial markers to accurately detect different stages of the process for nine dressing scenarios common to PWDs [22], clothing worn: correctly (shirt and pants); partially or on one limb, that is, one arm worn or one leg worn (shirt and pants); backwards with the back in front (shirt and pants); inside out (shirt and pants); and misaligned (for shirt only).

Before receiving specific instructions on how to perform each of the dressing conditions outlined in the study, participants were asked to retrieve two pieces of clothing from the drawer and to put them on in the manner they would normally perform at home. This step was included to identify whether any dramatic differences were identified because of the constraints of the dressing scenario instructions (not found to be the case) and for future analysis of the natural dressing process. As participants performed this “natural dressing” task, the chair was available to them, but none chose to use it.

Initially, the study incorporated a Kinect for event detection. Preliminary study determined that the Kinect could not reliably track participants’ skeletal actions because of occlusion by the garment during dressing. Use of the fiducial marker tracking was found to offer more effective tracking of garment orientation and position during dressing and was selected to both simplify and increase the reliability of garment recognition to assist the DRESS prototype in developing prompting and interventions to guide dressing.

Participants were then given the dressing conditions to be performed. The experimenter explained each step in the process using pictures (Figure 7). Pictures of the target dressing conditions were used instead of video (or specific descriptions of intermediate procedures) to minimize introduction of experimenter bias.

Participants were instructed that each trial would consist of the following steps: (1) wait for the experimenter to cue what dressing condition to perform and when to start; (2) pick the appropriate clothing item from the drawer; (3) don the garment in the way prescribed for the condition; (4) once completed, wait 3 seconds standing in front of the dresser saying slowly “DONE 1 2 3”; (5); if the condition involved an acted error to then engage in the DRESS prompted corrections; and (6) wait 3 seconds, again, saying slowly “DONE 1 2 3,” once the prompted dressing activity had been completed.

After completion, when prompted, the participant removed the clothing item off and gave it back to the experimenter. The experimenter would then orient the clothing (correctly, inside out, etc, as appropriate) and place the clothing back in the respective drawer to prepare for the next trial. Participants were reminded to complete each trial by wearing the clothing correctly in the manner they would normally prepare to leave their home and to use the chair when putting on or readjusting the pants. All participants performed each of the nine dressing conditions twice following a complete randomized block design. Finally, each participant session ended with the postsession survey.

Once we collected the data, we analyzed the DRESS prototype’s detection performance by comparing Indigo server recorded detection events (Table 1) with the expected detections for each of the six identified phases of the dressing process in each condition (first 2 title rows in Table 2). Phases of participants’ dressing actions included (1) Adjusting the clothing after being worn incorrectly spontaneously or by design (conditions with acted errors), (2) Putting the first limb on, (3) Transitioning between limbs, (4) Putting second limb on, (5) Transitioning to completion and adjustment, and (6) Completing the correct dressing process by standing in front of the camera.

Computer recordings of the trials were visually inspected to identify reasons for any missing or incorrect detections. Screen recordings included the video of the ps3view camera showing the participant’s action and the fiducial detection information (visible marker when detected; Figure 5, right), the experimenter’s actions (eg, setting, starting, and stopping the trial condition), and the computer’s time stamp.

Due to technical difficulties recovering data files for two trials, data were available for analysis for 108 of 110 experimental trials for the shirts and 86 of 88 trials for the pants.

## Results

### DRESS Prototype

**Figure 8:**
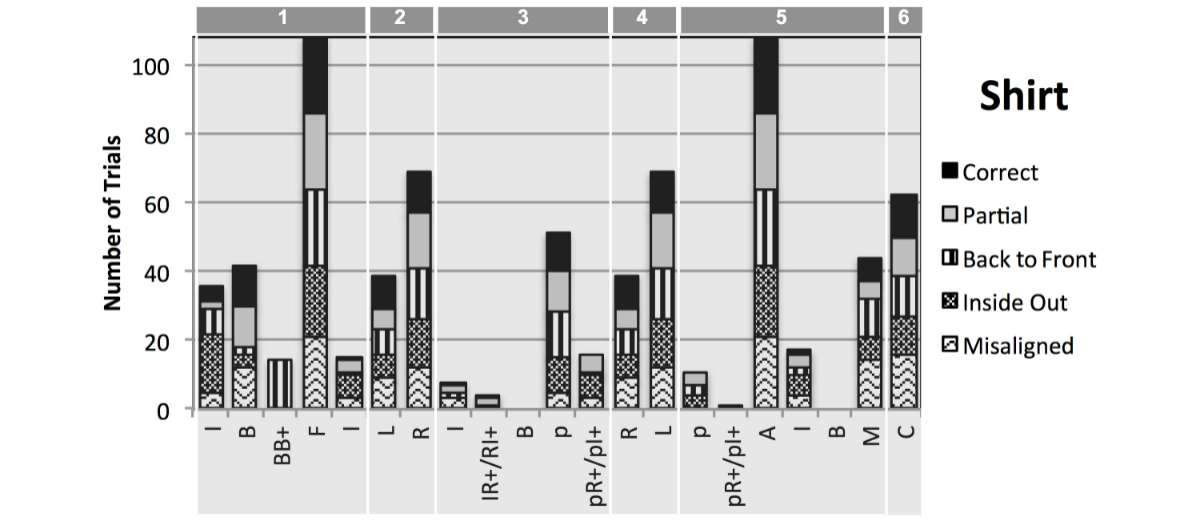
Shirt detection events indicate the number of trials (y-axis, maximum 108) for each recorded detection (x-axis), for each of the 5 conditions indicated by the filling pattern. Detection labels: I for Inside of clothing; B for back to front; BB+ for back to front detected more than twice in a row; IR+ or RI+ for sequence of inside then right or right and then inside detected more than once in a row; L for left side; R for right side; p for partial dressing; pI+ or pR+ for sequence of partial and inside or partial and right detected more than once in a row; C for correctly worn. Detections are presented for each of the 6 phases indicated on the top of the graph: (1) Preliminary error or adjustment; (2) 1st limb worn; (3) 1st limb to 2nd limb transition; (4) 2nd limb worn; (5) 2nd limb to completion transition; and (6) Correct completion.

**Figure 9:**
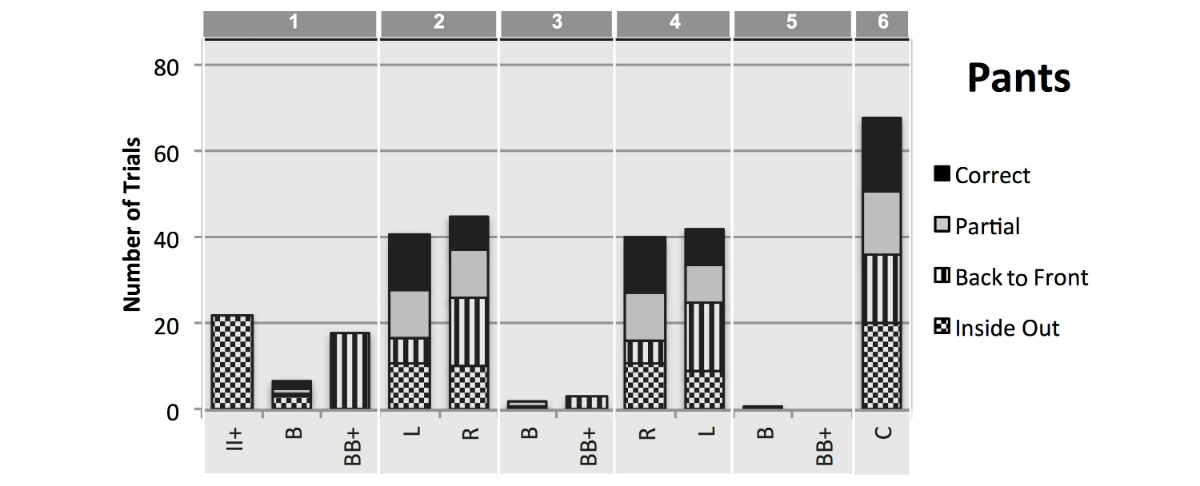
Pants detection events indicate the number of trials (y-axis, maximum 86) for each recorded detection (x-axis) for each of the 4 conditions indicated by the filling pattern. Detection labels: II+ for inside of clothing detected more than twice in a row; B for back to front; BB+ for back to front detected more than twice in a row; F for front side; L for left side; R for right side; C for correctly worn. Detections are presented for each of the 6 phases indicated on the top of the graph: (1) Preliminary error or adjustment; (2) 1st limb worn; (3) 1st limb to 2nd limb transition; (4) 2nd limb worn; (5) 2nd limb to completion transition; and (6) Correct completion.

Results indicated that the DRESS prototype was most reliable at reporting expected detections for acted errors of inside out pants and shirt in phase 1, followed by detection of each limb worn in phases 2 and 4 for both clothing items—missing only 4 out of 388 expected detections (see Table 2,Figures 8 and 9). The ability to recognize other missing detections varied across conditions. Furthermore, the DRESS prototype identified several initially unexpected detections, for example, recognizing the inside of the shirt or back of the pants during transition phases. On the basis of video analysis, the plausible detections that occurred most often among participants were identified as “expected detections” for each phase (presented in parenthesis, Table 2).

### Detection of Shirt

When participants were asked to wear the shirt correctly, we expected that the DRESS prototype would sporadically detect and record the inside (I) or the back of the shirt (B) before putting it on as it was removed from the drawer. We expected the front of the shirt (F) to be detected in the first phase, then, when one arm is worn (R/L in phase 2), when the other arm is worn (L/R in phase 4), to detect wearing of both arms (A), and finally correctly completing the dressing process by aligning and attaching the Velcro (C).

In the transition phases, we expected to also see the back of the shirt (B) or the inside (I) when the shirt was folded or moved around or above the person while dressing. Between donning the first and second arm, we expected to detect partial dressing (p) even at times when this was not the condition. Finally, we expected some misalignment (M) detections while adjusting preceding completion.

**Table 2:** Detections for each of the six identified phases for pants and shirt conditions. Letters indicate detection labels, and parentheses are used to indicate the expected detection events. Italicized fonts indicate unexpected detection results where the number shows the frequency (with positive and negative representing undesirable and missing expected detections, respectively).

Dressing Errors		Dressing phases					
		1	2	3	4	5	6
		Preliminary error or adjustment	1st limb worn	1st limb to 2nd limb transition	2nd limb worn	2nd limb to completion transition	Correct completion
**Pants**							
	Correct	(B^a^)	R^b^/L^c^	—	L/R	—	−*4C*^d^
	Back to front	−*3B*	R/L	*4B*	L/−*1R*	—	−*6C*
	Inside-out	I^e^,(B)	R/L	*1B*	−*1L*/R	—	−*2C*
	Partial	(B)	R/L	—	−*2L*/R	*1B*	−*7C*
**Shirt**							
	Correct	F^f^,(B),(I)	R/L	(p^g^),(I),(R/L)	L/R	A^h^,(M^i^),(I),*(1p)*	−*10C*
	Back to front	F*,−6B*,(I)	R/L	(p),(I),(R/L)	L/R	A,(M),(I),*(3p)*	−*10C*
	Inside-out	F,(B),I	R/L	(p),(R/L)	L/R	A,(M),(I)	−*10C*
	Misaligned	F,(B),(I)	R/L	(p),(I),(R/L)	L/R	A,*−7M*,(I)	−*5C*
	Partial	F,(B),(I)	R/L	−*5p*,(I),(R/L)	L/R	A,(M),(I),*(4p)*	−*11C*

^a^B: detection label for back side.

^b^R: detection label for right arm/leg.

^c^L: detection label for left arm/leg.

^d^C: detection label for worn correctly.

^e^I: detection label for inside part.

^f^F: detection label for front side.

^g^p: detection label for partial dressing.

^h^A: detection label for both arms of the shirt.

^i^M: detection label for Velcro unevenly fastened or misaligned for shirt.

We found that the DRESS prototype was completely reliable at detecting initial donning of the shirt (F) and was likewise completely reliable in phases 2 and 4 when the participant put on each of the two arms. Figure 9 shows the detections expected for each condition in each phase of the testing, and the black bars show the counts of detections in the correct condition. The DRESS prototype missed correct dressing completion detections in 10 of 22 cases. Reasons included limitations in the detection of markers, but also included the threshold of Velcro markers being too small (eg, if participants moved too far from the camera) and when participants did not correct the Velcro misalignment even when prompted to do so. Providing a mirror to check correctness and alignment might successfully alleviate this issue.

With respect to shirt error conditions, the DRESS prototype was most reliable in detecting inside out errors, missing only a single detection. In one occasion, the participant appeared confused about the orientation of the shirt and turned it inside out several times before completing donning, resulting in an unexpected but accurate condition recognition. Other conditions resulted in similar detection reliability, missing 5, 6, and 7 detections for partial, back to front, and misalignment conditions, respectively (see Table 2).

Unexpected detections included partial (P) detections after completing wearing of the second arm just before completion of donning (phase 5). Video inspection revealed that the unexpected detections were primarily because of lengthy adjustments by the subjects before closing the shirt. Adjustments included opening and closing the shirt several times to bring the two parts of the shirt together; holding the neck or Velcro occluding the markers; and for females, adjusting hair and occluding the markers while completing the dressing process. In one case, the shirt was too large for the participant, resulting in folds that impeded marker detection.

### Detection of Pants

When participants were asked to don pants while seated, we expected the following detections through the phases of the process: sporadic back of pants detection when adjusting before putting the pants on (B in phase 1); right or left leg worn (R/L in phase 2); then other leg worn (L/R in phase 4); and finally that donning pants was correctly completed (C in phase 6). The DRESS prototype successfully recognized all the detections except phase 6, where completion detection was missed 5 out of 22 trials (see Table 2 and black bars in Figure 9).

In terms of detecting acted errors in phase 1, the DRESS prototype was most reliable in detecting when pants were donned inside out (100%) and only missed three back to front detections (see phase 1 of Table 2 and stripes and dotted bars in Figure 9). For the partial dressing condition, the DRESS prototype failed to correctly record partial dressing, Video examination indicated that partial dressing was detected in some occasions when the middle to upper fiducials on one pant leg were detected while the fiducials of the other pants leg were not. Data inspection indicated that no completed detections were recorded for only partial dressing. However, because of folding while pants are partially worn during donning, detection of partial dressing remains challenging.

Missing detections were found to occur as subjects put on the second leg (L/R phase 4) and upon correct completion of the dressing process (C phase 6). Visual inspection of the videos indicated that missing detections were because of (1) Inability of the camera to see markers, (2) Suboptimal position of the participant with respect to the camera (tilted, too close, too short), (3) Occlusion by folds in the cloth when the clothes were too large for the participant, (4) Failure of the Indigo server to recognize the visible markers or record the detection events on time, and (5) Participant donning the clothing too quickly for the marker to be captured and recognized.

Unexpectedly, the DRESS prototype detected back to front orientation in six trials during transition phases during donning (3 and 5). Several events related to clothing adjustment were found to account for these detections, including (1) Turning the pants around while wearing one leg; (2) Holding the pants in front of the camera long enough in an orientation that the DRESS prototype incorrectly detected wearing one leg, affecting the interpretation of the following detections; (3) Taking the pants off after completing the partial scenario; (4) Readjusting the pants in a manner in which the back markers were visible momentarily; and (5) While adjusting the pants after turning them inside out in the inside-out dressing condition.

## Discussion

The main findings of this capabilities study are that the DRESS prototype incorrectly identified 10 of 22 cases for shirts and only 5 of 22 cases for pants. Through this process, we identified several significant opportunities to improve the DRESS prototype’s reliability, such that it can provide substantive support for PWDs engaged in dressing activities in subsequent in-home trials.

Intelligent dressing systems that support PWD need to understand and adapt to the complex and dynamic processes involved in donning each clothing item. These systems must also be able to adapt to challenges that may occur in these processes that can lead to incorrect dressing.

The DRESS prototype identifies states within the dressing process through its ability to detect fiducial markers on clothing items. Marker detection is used to infer context and improve the accuracy of the DRESS prototype. Additional trials are needed to further determine optimal positions for marker placements to minimize potential interference and to ensure that the DRESS prototype can accurately detect and respond to a PWD’s dressing activities.

Improving the accuracy of marker detection is only a part of the solution. To provide efficient feedback and guidance to the user, DRESS enables caregivers to customize its support so as to foster a PWD’s personalized dressing sequence. Awareness of this sequence of actions may help DRESS in determining dressing progress and generating prompts and guidance. As dementia is often a progressive condition, the data generated in the tracking of progress and provision of these prompts may be useful in the assessment of cognitive decline overtime. At times when PWDs’ dressing process has stalled, caregivers will receive an alert prompting them to intervene; as an alternative to responding to these alerts, in person, we plan on providing caregivers with tools to provide remote intervention via the DRESS prototype. Using a mobile phone, caregivers will be able to control the iPad and iPods on the drawers to provide voice and visual prompts, a technique known in the field of human-computer interaction as the “Wizard of Oz” method.

We have discussed laboratory testing of our DRESS prototype, integrating sensors and fiducial tracking to identify and guide the dressing process. However, successful deployment in the homes of PWD is more complex. For example, we must address the physical and cognitive differences between the population used in our study and PWDs. We plan to assess potential participants for our initial home trials to ensure that their physical capabilities will not encumber either their dressing activities or use of DRESS. In our initial home trials with PWDs, we will exclude individuals with limited physical capabilities (an issue that might encumber either their dressing activities or use of DRESS) to focus on the systems’ ability to attend to the cognitive differences between the population used in this study and PWDs. This will enable our investigative efforts to focus on fine-tuning the DRESS prototype in ways that will appropriately attend to the cognitive needs of PWDs. We will pay particular attention to determining how the DRESS prototype prompts and the diverse nature of individuals’ home environments might alter the detecting process and results.

Additional efforts will involve use of more acceptable markers, for example, using infrared inks to provide “invisible” markings on PWD’s existing clothes. Although these “invisible markers” cannot be seen by humans, they can still be detected by the reacTIVision system. Members of our team are also exploring the potential for using machine-vision to train and recognize patterns inherent to individual PWD’s existing clothing.

As part of the home deployment phase, we will also conduct cost-benefit analyses to determine the economic value of DRESS. Although it is too early to predict final product expense or initial markets segments (domestic, group homes, etc) a rough cost or benefit estimate can be considered. Assume a conservative rate of US $20/day to represent the combined value of increased PWD independence and reduced caregiver stress and effort, approximately US $600/month. In 2 to 4 months, a system cost of US $1200 to US $2000 would be recouped. Subscription plans rather than purchase might also make such systems broadly affordable.

Our study has shown that the DRESS prototype can detect clothing orientation and position and infer current state of dressing using a combination of sensors, intelligent software, and fiducial tracking. The DRESS prototype demonstrates a promising step toward automated dressing support to assist PWDs in maintaining their independence and privacy, while potentially providing their caregivers with much needed respite. We have identified several opportunities for improvement of the DRESS prototype. We plan to improve the markers, making them less obtrusive by making them “invisible” and/or training recognition systems to detect the natural patterns present in PWDs’ existing clothes. These endeavors aim to optimize the overall detection of dressing status, as we embark on the next stage of our research agenda—deployment of the next iteration of the DRESS prototype in the homes of PWDs.

## Abbreviations

ADL: activities of daily living
PWD: person with dementia
RFID: radio-frequency identification
